# Identifying Factors of User Acceptance of a Drone-Based Medication Delivery: User-Centered Design Approach

**DOI:** 10.2196/51587

**Published:** 2024-04-30

**Authors:** Franziska Fink, Ivonne Kalter, Jenny-Victoria Steindorff, Hans Konrad Helmbold, Denny Paulicke, Patrick Jahn

**Affiliations:** 1 Translation Region for Digitalised Healthcare Department of Internal Medicine, Faculty of Medicine, University Medicine Martin-Luther-University Halle-Wittenberg Halle (Saale) Germany; 2 Health Service Research Working Group | Acute Care Department of Internal Medicine, Faculty of Medicine, University Medicine Martin-Luther-University Halle-Wittenberg Halle (Saale) Germany; 3 Department of Economics Anhalt University of Applied Sciences Bernburg Germany; 4 Department of Medical Pedagogy Akkon University of Human Sciences Berlin Germany

**Keywords:** human-drone interaction, medical supplies, participative research, user-centered design, technology acceptance

## Abstract

**Background:**

The use of drones in the health care sector is increasingly being discussed against the background of the aging population and the growing shortage of skilled workers. In particular, the use of drones to provide medication in rural areas could bring advantages for the care of people with and without a need for care. However, there are hardly any data available that focus on the interaction between humans and drones.

**Objective:**

This study aims to disclose and analyze factors associated with user acceptance of drone-based medication delivery to derive practice-relevant guidance points for participatory technology development (for apps and drones).

**Methods:**

A controlled mixed methods study was conducted that supports the technical development process of an app design for drone-assisted drug delivery based on a participatory research design. For the quantitative analysis, established and standardized survey instruments to capture technology acceptance, such as the System Usability Scale; Technology Usage Inventory (TUI); and the Motivation, Engagement, and Thriving in User Experience model, were used. To avoid possible biasing effects from a continuous user development (eg, response shifts and learning effects), an ad hoc group was formed at each of the 3 iterative development steps and was subsequently compared with the consisting core group, which went through all 3 iterations.

**Results:**

The study found a positive correlation between the usability of a pharmacy drone app and participants’ willingness to use it (*r*=0.833). Participants’ perception of usefulness positively influenced their willingness to use the app (*r*=0.487; TUI). Skepticism had a negative impact on perceived usability and willingness to use it (*r*=−0.542; System Usability Scale and *r*=−0.446; TUI). The study found that usefulness, skepticism, and curiosity explained most of the intention to use the app (*F*_3,17_=21.12; *P*<.001; *R*^2^=0.788; adjusted *R*^2^=0.751). The core group showed higher ratings on the intention to use the pharmacy drone app than the ad hoc groups. Results of the 2-tailed *t* tests showed a higher rating on usability for the third iteration of the core group compared with the first iteration.

**Conclusions:**

With the help of the participatory design, important aspects of acceptance could be revealed by the people involved in relation to drone-assisted drug delivery. For example, the length of time spent using the technology is an important factor for the intention to use the app. Technology-specific factors such as user-friendliness or curiosity are directly related to the use acceptance of the drone app. Results of this study showed that the more participants perceived their own competence in handling the app, the more they were willing to use the technology and the more they rated the app as usable.

## Introduction

### Background

The health care system faces challenges such as a rural exodus, aging populations, and increasing shortages in the health care workforce; drones have the potential to increase the efficiency and capacity of the health care system [1]. The COVID-19 pandemic intensified the demand for new logistic solutions, such as fast and contactless delivery strategies [2]. It is also important to understand the attitude of the civilian population and public opinion on the use of drones [3]. In this vein, delivering medications with drones is the most identified application in health care [1,4]. There are a few studies showing that usability, lack of user skills and expertise, and negative perceptions affect user acceptance and hinder drone use [1,5,6]. Therefore, it is particularly important that all user groups are involved at an early stage. Furthermore, after a recent scoping review showed that there were no empirical studies on user acceptance of drone-based medication delivery [7], we could only find 1 study in Asia that investigated user acceptance among health care professionals in the delivery of drone-based medication [8]. The successful application of technology is predominantly determined by the type and extent of acceptance [9,10]. Acceptance in this context refers to the positive acceptance decision of an innovation by its users, which is described in the technology acceptance model (TAM; perceived usability, usefulness, immersion, and accessibility: TAM 1) [11,12]. It proposes that users tend to use a technology when they believe it will help them to perform a better job (perceived usefulness) and when they believe that the system can be handled without effort (perceived ease of use). These variables were found to correlate with the intention to use, wherein usefulness was substantially more strongly related to frequency of use than ease of use. Nevertheless, both are strong correlates of user acceptance and should not be ignored by designing and implementing successful technologies [12]. In other words, the greater the benefit of a technology and the simpler its usability, the more the users are willing to use the new system. However, some more variables that affect user acceptance such as social influences (subjective norm, image, and voluntariness), cognitive instrumental processes (job relevance, output quality, and result demonstrability; TAM 2), and psychological foundations (self-efficacy, external control, playfulness, anxiety, enjoyment, and usability; TAM 3) can be stated [13,14]. Thus, Peters et al [15] argued that this model alone does not indicate whether people would actually use a technology. In this context, basic human needs according to Ryan and Deci [16,17] and Deci and Ryan [18] play an important role. Following the Basic Psychological Need Theory [17], the more the interaction with the system satisfies basic psychological needs, the more the users will engage with a technology. Ryan and Deci [16,17] and Deci and Ryan [18], defined 3 basic psychological needs in their self-determination theory that are crucial to whether a person is proactive and engaged or passive and demotivated. These needs include competence (ie, feeling capable), relatedness (ie, feeling connected to others), and autonomy (ie, feeling self-determined). On the basis of this, Peters et al [15] defined the Motivation, Engagement, Thriving in User Experience (METUX) model, which can be used for the evaluation and iterative design process of technologies to increase motivation, engagement, and well-being. In this case, the 3 basic needs are mediating variables between a technical product and the well-being of the users. This implies that as autonomy increases, engagement increases; as competence increases, motivation increases; and as relatedness increases, well-being increases.

### This Study

**Figure 1:**
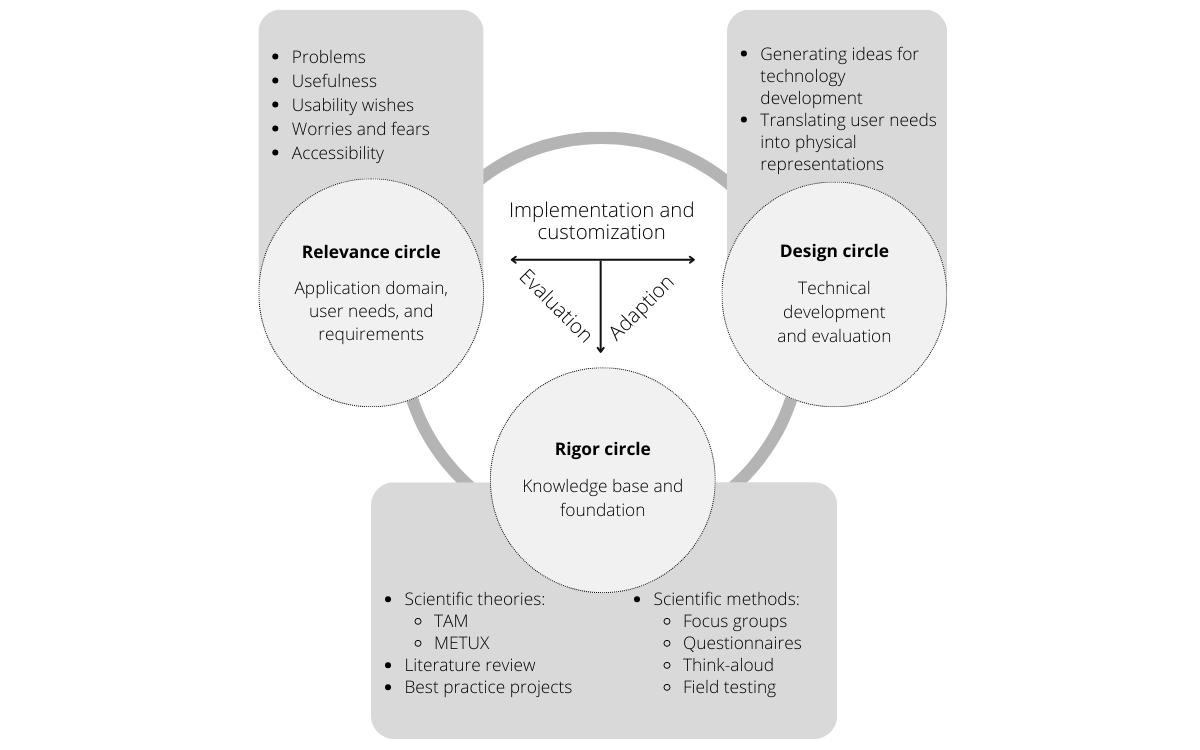
Implementation circle. METUX: Motivation, Engagement, Thriving in User Experience; TAM: technology acceptance model.

Acceptance building is a process that starts before the initial contact with the innovation and continues into the application phase, which was addressed within the pharmacy drone study (Apotheken-Drohnen-App; ADApp) [19]. This study represents a section of the whole ADApp project by investigating factors that are associated with the user acceptance of a drone-based medication delivery to be able to derive practice-relevant orientation points for participatory technology development (for apps and drones). Stephan et al [7] described that little attention is paid to the design phases of drones including the delivery process. Thus, this study used a mixed methods design and followed the methodological guidelines of the cocreative user-centered design proposed by Farao et al [20]. User-centered design is used to help consider the context of technologies as well as their implementation consequences at the design stage (Figure 1) [20,21]. Implementing technologies without user involvement may compromise the desired outcome, which in turn can lead to unmet health goals and adverse outcomes [20,22,23]. It is an evidence-based approach that involves users in developing technologies and prioritizes their needs [20,24]. In contrast to classical user-centered designs, this study used a controlled design for the first time. Traditionally, small sample groups are observed or interviewed or participate in usability tests during the testing and development phases of new technologies (usually operationalized through the think-aloud [TA] method and questionnaires). These are important approaches to get insights into key needs of the target population [25]. However, repeated measurements cause a change in the meaning of test scores, which makes it difficult to compare repeated measures. In a measurement perspective, it can be considered as bias in the measurement of change [26]. It can be inferred that conducting repeated measures with the same sample group may alter their attitudes, expectations, and behaviors in interacting with the technology. This could potentially influence their acceptance of the technology, as they become aware that the technology will adjust to their needs. Moreover, they are not unfamiliar with dealing with the app, which might influence user acceptance as well. This is indeed the purpose of a user-centered design, but it loses insights into perspectives of inexperienced users without a concrete expectation about the changes in technology after giving feedback to it. By integrating a control group (called the *ad hoc* *group*), this study aimed to investigate whether the assumption of the *core group* (ie, the same sample group at all measurement time points) is generalizable to a broader population. In this regard, a second purpose of this study was to answer the question of whether user acceptance differs between the core and ad hoc groups.

## Methods

### Study Design

The monocentric ADApp study aimed at an iterative and cocreative development of a pharmacy drone app with multiple measurement time points (Table 1). As this study design was embedded in the broader ADApp project, it was preceded by 2 research steps. As a first step, we conducted a scoping review of experimental studies examining the interaction between humans and drones during the delivery of drugs and defibrillators to identify research gaps and explore the scope of research activities [7]. In the next step, problems, needs, and requirements of the users were identified and concrete scenarios were outlined, which were necessary for the implementation of a first demonstrator of the app [27]. At this point, we decided to use focus groups instead of individual interviews because it allows participants to respond to each other’s answers and gives us the most information. In this study, we conducted 3 iteration loops, where we tested the app demonstrators a well as the entire ADApp flow from order to delivery at an airport along with the user groups. The iterative process is one of the central features of the study and will be discussed in detail in the Study Setting section.

**Table 1:** The Apotheken-Drohnen-App design.

Goal	Methods	Participation	Time point	Reference
Knowledge	Scoping review	N/A^a^	August 2021	Stephan et al [7], 2022
Needs	Focus groups	User groups	October 6, 2021	Fink et al [27], 2023
Evaluation of functionalities	Questionnaires and think-aloud	First and second iteration loop: core group; 2 ad hoc groups	July 3, 2022	This study
Test flights	Questionnaire, think-aloud, and focus groups	Third iteration loop: core group; 1 ad hoc group	October 2022	This study

^a^N/A: not applicable.

To address potential biasing effects owing to a response shift through repeat interviews with the core group, a total of 3 ad hoc groups were acquired in this study. At each of the 3 development steps, a new and naive ad hoc group was used. This unique approach of adding ad hoc groups as control groups also allows for the generalizability of key needs identified in the development process [25].

### Study Setting

Our prior work showed that a pharmacy drone app, for example, must be lean and simple; must facilitate the user’s performance (eg, software integration, shipment tracking, and plannability); must enable control (eg, handover identification and data sovereignty); and must include consultation and reconciliation features. The most frequently discussed problems associated with drones were the physical contact with the drone and the drone’s noise [27]. Following the focus groups, the developers designed a demonstrator of the app including communication features (eg, an extra text field for communication with the pharmacy), plannability features (eg, time slots for drone delivery), shipment tracking, and features for enabling handover security, which was tested in 3 iteration loops with users. All loops were conducted separately per the core and ad hoc groups.

**Figure 2:**
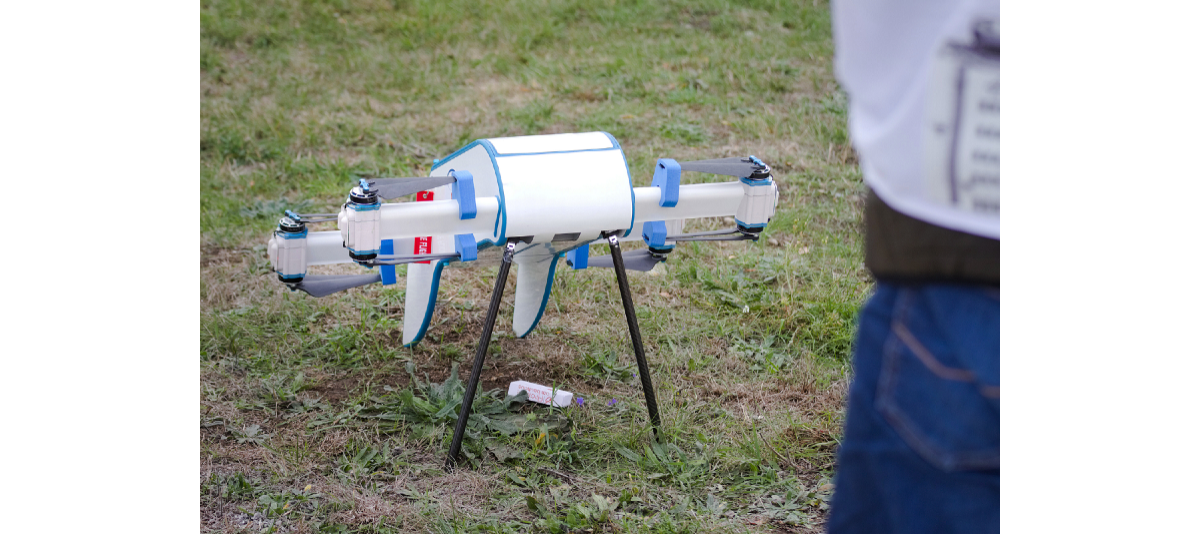
Drone landing.

**Figure 3:**
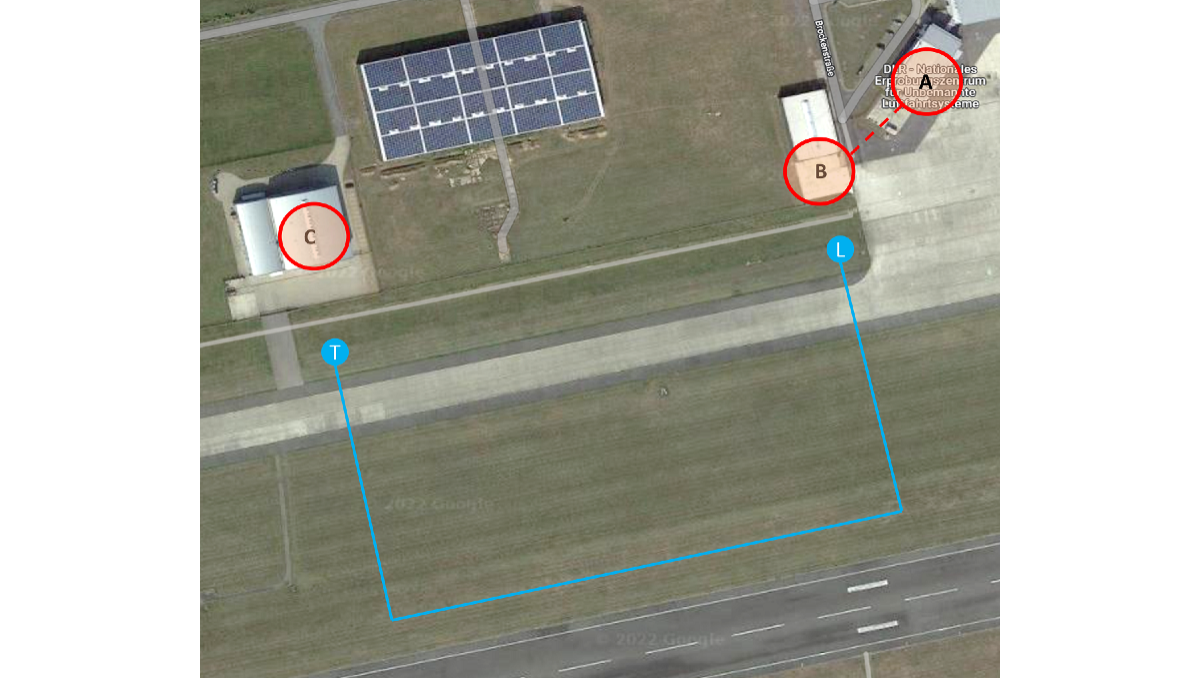
Airport landing zone. (A) Order location, (B) reception location, and (C) drone starting point. L: landing; T: takeoff.

In the *first iteration loop* (conducted between March and April 2022), the usability of the app was reviewed. The app should be as easy as possible to use and allow users to create an account, add details such as delivery location, or submit an electronic prescription. The first iteration loop aimed at evaluating how intuitive the app is. For this purpose, users were shown the app and asked to think aloud while using it—without any introduction. For each participant, one experimenter prepared a protocol for taking notes. Finally, the prototypes were queried by using additional questionnaires: the Technology Usage Inventory (TUI) assessment was used to assess the intention to use, and the System Usability Scale (SUS) was used to measure usability. The basic psychological needs such as competence, autonomy, and connectedness were identified at the task level through the Technology-based Experience of Need Satisfaction (TENS) Task. After the first iteration loop, the demonstrator was adapted according to user feedback. The *second iteration loop* (conducted between June and July 2022) was dedicated to the design of the prototype and its technical development and evaluation. The decisive factor was how intuitive the design is and whether the tasks of the app are adequately represented. The procedure was the same as that in the first iteration loop, with the exception that instead of the TENS Task, basic psychological needs were queried via the TENS Interface. To gain deep insights into the participants’ thoughts while using the technology, the core and ad hoc groups were again asked to think aloud while using the app. After receiving the second round of feedback from participants, a third demonstrator was developed. However, the *third iteration loop* (conducted in October 2022) aimed at testing the overall process starting with registration, setting the delivery location, submitting a prescription, and actually receiving a delivery. As focus groups showed concerns about injuries with the drone, this study wanted to test the handover via a winch, a parachute, or dropping to reduce physical contact with the drone, but unfortunately, owing to regulations and restrictions, testing the handover was not possible [27]. Thus, the drone had to land (Figure 2). Therefore, we divided participants into 2 groups: one group tested the app, whereas the other group talked about different handover scenarios and looked at the drone from close up. After each round, the groups were swapped. Similar to that in the first and second iteration loops, the intention to use and usability was queried using the TUI and SUS. To gauge the degree to which the technology fulfills users’ needs in terms of behavior, participants completed the Basic Psychological Need Satisfaction and Frustration Scale (BPNSFS). Again, the participants were instructed to think aloud while going through the tasks in the app to gain more insights into the functionality of the app. After participants submitted their prescription, they went to the location in the airport where the drone was set to land (Figure 3). After landing, participants took the *medication* (a bag of gummy bears) out of the drone. After the testing, a short discussion was held with all participants to sum up their impressions.

### Participants

Participants were chosen to represent potential user groups for both the drone-based medication delivery service and the supply chain, based on their respective role characteristics: physicians, nurses, pharmacists, and interested users, especially patients with COVID-19 and relatives of patients who need palliative care. Participants of the ad hoc group were recruited with the support of the Merseburger Innovations- und Technologiezentrum (innovation and technology center) and the ADApp project team. They were contacted via email or telephone. Core group participants were recruited from the focus group that was conducted in October 2021 [27]. A total of 3 pharmacists, 2 nurses, 3 general practitioners, and 1 patient with COVID-19 were recruited from the focus groups. Owing to the underrepresentation of interested users or patients, 2 more participants were recruited with the support of the ADApp project team.

All participants were informed about the general aim and reasons of the study and the procedure. They gave written informed consent before starting the iteration loops.

### Ethics Approval

The ADApp study was approved by the Ethics Committee of the Martin-Luther University Halle-Wittenberg (protocol code 2021–069; date of approval May 6, 2021).

### Measures

#### Quantitative Measures

##### SUS Score

The SUS measures the user’s subjective perception of the usability of a system. It is technology independent, that is, it can be used for a wide range of systems and technologies [28-30]. Overall, 10 items were divided into 5 positively and 5 negatively directed statements, each represented on a 5-point Likert scale. The answers provided the SUS item score, which were then converted into the SUS overall score. The overall score ranges from 0 to 100. To calculate the SUS overall score, the first step was to calculate the raw score minus 1 for all odd items, whereas the raw score of 5 was subtracted for the even items. For example, if for item 1, the raw score is 4, the result is a score of 3 (4-1). If for item 2 the raw score is 2, the score is 3 (5-2). In the next step, the calculated scores are summed and multiplied by 2.5 [31].

Systems can be considered usable if they achieve the benchmark score of ≥68 [29,31]. In preliminary works, an adjective scale was developed for a more comprehensible classification of the SUS score, which ranges from outstanding (score 90-100) to very poor (score 0-34) [32].

##### TUI Assessment

The TUI assessment [33] aims to measure the intention to use and is based on the TAM [12]. The intention to use a technology is a comprehensive construct based on a variety of explanatory factors. As suggested in the TAM 2 and TAM 3, the TUI considers technology-specific factors and psychological factors. The TUI therefore supplements the classic technology acceptance factors of the TAM 1, such as perceived usability, usefulness, immersion, and accessibility (technology specific) with important psychological constructs, such as technology anxiety, curiosity, interest, and skepticism. The items, with the exception of the technology anxiety and interest scale, are related to a specific technology. Each scale consists of 3 to 4 items, each of which is to be rated on a 7-point Likert scale. The ninth scale measures the intention to use a specific technology with 3 items on a visual analog scale (each 100 mm). In total, the TUI consists of 33 items and has a modular design so that individual scales can be excluded and item formulations can be adapted to the circumstances (eg, concrete technology names). With the exception of the “immersion” scale, all scales were used this study. All answers of a scale were summed to a sum value. It starts with 1 as the lowest expression of the construct and goes up to 21 (3 items) or 28 (4 items) as the highest expression, depending on the number of items. For the intention to use scale, the distance from the right end point (full rejection) to the answer cross on the line are measured. The distance in mm is determined for all 3 items and summed. The maximum scale value to be achieved is 300. The determined scale sum values are converted into standard values (stanines). The stanines reach from 1 (strongly below average) to 9 (strongly above average) [33].

##### METUX Model

The “pure” usability does not necessarily predict higher use of a technology [34]. Although the SUS questionnaire focuses on usability, the TUI questionnaire also includes psychological factors. However, both neglect basic psychological needs, which are addressed in the METUX model. It aims to optimize engagement, motivation, and well-being of technologies in iterative design processes [15]. Within the model, different spheres of experience were assumed to influence well-being: interface (ie, interacting with the technology), tasks (ie, engaging with the technology), behavior (ie, the relation to the overarching technology-supported behavior), and life (ie, the overall experience outside and beyond the technology). There are different questionnaires for measuring the basic psychological needs in different spheres. The spheres adoption, interface, tasks, and behavior were tested within this study using the Autonomy and Competence in Technology Adoption Questionnaire for the first adoption process, the TENS [15] for interface and task, and the BPNSFS [35,36] for behavior. The sphere “life” was not relevant for this study. The Autonomy and Competence in Technology Adoption Questionnaire addresses the question of why people use a technology and to what extent they experience themselves as competent to use it. It consists of 2 parts: the first, self-regulation, includes 12 items; and the second, perceived competence, includes 2 items, which are represented on a 5-point Likert scale. The goal of the TENS Interface and Task questionnaires is to assess the extent to which direct interaction (via interface) with a technology and engagement in technology-specific tasks satisfies the basic psychological needs for autonomy, competence, and relatedness [15]. In the TENS questionnaires, the items are each assigned to the basic needs of competence, autonomy, and relatedness but are presented randomly in the questionnaire. All items were equally weighted, summed, and averaged per basic need. The TENS Interface questionnaire consists of 15 items with 5 items per need, whereas the TENS Task questionnaire consists of 12 items with 4 items per need. The items are each represented on a 5-point Likert scale. As the TENS questionnaires are only validated for English-speaking countries so far, a linguistic validation of the questionnaires was conducted. For this purpose, the questionnaires were translated into German by an interpreter whose native language is German and who is fluent in English. Nevertheless, both the TENS questionnaires are not standardized for the German population that has to be considered when interpreting the results.

The BPNSFS is intended to assess the extent to which a technology improves need satisfaction in relation to the behavior the technology is intended to support [15]. The BPNSFS measures the satisfaction and frustration of the basic psychological needs of autonomy, competence, and relatedness. This includes a balanced combination of subscales for satisfaction and frustration. This distinction is necessary because the absence of need satisfaction does not equate to frustration of the same [17,35]. On the basis of the original questionnaire, Heissel et al [36] identified 6 different, but intercorrelating, factors with 4 items each for the German version of the BPNSFS: Autonomy Satisfaction, Autonomy Frustration, Competence Satisfaction, Competence Frustration, Relatedness Satisfaction, and Relatedness Frustration. Each response is represented on a 5-point Likert scale. The evaluation of the BPNSFS can be handled differently; for this study, the items per basic need are summed, and the 12 items of the subscales satisfaction and frustration are summed. There exist several adaptations of the BPNSFS, which have been validated and subjected to reliability testing. These adaptations include language translations, adjustment for age (children or adults), domain (sports, work, and romantic relationships), and clinical status (HIV, intellectual impairment, and chronic pain). However, a questionnaire related to technological aspects does not yet exist. Thus, for this study, the German version of the BPNSFS was used and minimally adapted according to the wording.

#### Qualitative Measures: TA Method

TA has traditionally been used in psychology and education to identify cognitive processes that occur internalized in the context of problem-solving [37]. In the context of technology development, TA is equally used to gain deep insights about thinking while using a technology [38]. The advantage of the method is to capture problems and solutions as the technology is being used, as retrospective surveys can lead to incomplete information about the problems of a technology. This means TA is helpful in tracing user thinking strategies [39,40]. For this purpose, participants were instructed to think aloud constantly while using the demonstrator. If participants stopped thinking aloud, they are reminded by the experimenter to continue speaking aloud [38,39]. For understanding problems and solutions, we did not explain how the demonstrator is supposed to work. Instead, we asked them to experience the app with little direction by explaining the task they had to do: registration, set delivery location, and submit recipe [41]. Thus, during the TA situation, it was important that the experimenter interact with participants as little as possible to prevent interference with the users’ thoughts [39]. During the TA situations, the statements of the participants were digitally recorded (audio recordings) and transcribed afterward. Moreover, experimenters prepared protocols for making notes and describing events that are not verbally made by participants but important for analyzing. For example, if a participant said, “That is confusing,” the experimenter protocoled what exactly was confusing [42].

### Data Analysis

#### Quantitative Analyses

Analyses were performed with the statistical program SPSS Statistics (version 25; IBM Corp). Bivariate correlation analyses were performed to assess the association between different user acceptance measurements of usability (SUS score), intention to use a technology (TUI factors using raw values), and psychological needs (the TENS Task and Interface as well as the BPNSFS using raw values). To investigate which factors are associated with the intention to use the pharmacy drone app, hierarchical regression analyses were performed among TUI factors as well as among all measurements. To answer the question whether TUI and SUS factors differ between the core and ad hoc groups, 2-tailed *t* tests were performed. Owing to the nature of the study design (small sample sizes), statistical analyses should not be overinterpreted; thus, the results of questionnaires were also analyzed descriptively according to the improvement of acceptance level of SUS and TUI scores.

#### Qualitative Analyses

The transcripts were analyzed according to the event sampling method of Berelson [43], where an utterance represents an event. Utterances are defined as a complete sentence, a sentence fragment, or any sequence of speech separated in time (eg, a pause of 2 seconds) or semantic (eg, a change in content) [39,44]. Utterances were analyzed by referring phrase analysis [39]. First, all nouns and noun phrases were identified, and utterances with the reference concept name were coded by the first author (FF). This coding shows which concepts the participants focused on during the task. After concepts have been identified, these concepts were defined by the investigator (Tables 2 and 3). The resulting coding scheme was used to code the statements of the participants. Another researcher (JS) who was familiar with the analyses analyzed randomly selected portions of transcripts (20%) to determine if there was a match. In case of disagreement, discussions were held between the 2 examiners until a consensus was reached. Cohen κ [45] was computed for all variables. The interrater reliability was κ=0.654 (*P*<.001) with a substantial agreement [46].

**Table 2:** Examples of the coded concepts.

Coded concept	Segment
Value and problem	“What is stupid now is this field. It does not disappear.” (Doctor, aged 48 years)
Proposal	“Would be nicer: ‘Your location and address has been confirmed.’” (Nurse, aged 51 years)
Value and conceptuality	“That black sign that irritates.” (Patient, aged 55 years)
Status	“Now I have uploaded this successfully.” (Pharmacist, aged 35 years)
Ambiguity	“Do I have to register again now?” (Nurse, aged 49 years)

**Table 3:** Definitions of coded concepts.

Concept	Definition
Value	Rating of usefulness, importance, or worth
Status	Information indicative of status or self-instruction
Problem	Technical inconvenience requiring action
Ambiguity	Incomprehensibility of the process, operation, or handling
Conceptuality	System of terms or concepts
Proposal	Recommendation for technical implementation

The experimenters who prepared the protocols during the iteration loops were asked to evaluate the accuracy of the definitions of coded concepts to ensure that no undefined concepts remained. After all utterances were coded, concepts were summarized for groups. The results were arranged in a table per task and discussed with the ADApp team to derive practice-relevant orientation points. To rank the participant’s points, we classified the concepts according to criteria within the ADApp team. The criteria helped us to evaluate the relevance of the concepts for developing the technology. We defined 4 criteria: safety, risk in the delivery, optimization potential, and outside the capabilities (Multimedia Appendix 1).

## Results

### Participants

For the 3 iteration loops, between March 2021 and October 2022, we collected data from 18 participants (mean age 43.08, SD 12.44; range 25-65 years). Owing to the relatively large amount of time required, not all participants of the core group could always participate in the iteration loops. In total, 6 participants took part in the first core group (1 general practitioner, 2 pharmacists, 1 patient, and 2 nurses); 3 participants took part in the second core group (1 nurse, 1 pharmacist, and 1 patient); and 4 participants took part in the third core group (2 nurses, 1 pharmacist, and 1 patient). Although, we tried to balance the 2 groups, it was not always possible to equalize the core and ad hoc group. Four participants took part in the first ad hoc group (1 general practitioner, 1 nurse, and 2 relatives of patients who need palliative care); 4 participants took part in the second ad hoc group (3 patients and 1 nurse); and 4 participants took part in the third ad hoc group (1 nurse and 3 patients). Table 4 provides detailed demographics.

**Table 4:** Participants’ demographics.

Characteristics		Ad hoc group 1 (n^a^=4)	Ad hoc group 2 (n=4)	Ad hoc group 3 (n=4)	Core group 1 (n=6)	Core group 2 (n=3)	Core group 3 (n=4)	All (n=18)
Age (years), mean (SD; range)		50.25 (3.59; 47-55)	47.25 (4.42; 43-52)	50.75 (16.19; 31-64)	39.17 (15.45; 25-65)	37.00 (11.53; 26-49)	34.5 (10.97; 26-49)	46.00 (12.19; 25-65)
**Gender (female)**								
	Values, mean (SD)	1.00 (0.00)	1.25 (0.50)	1.25 (0.50)	1.5 (0.55)	1.67 (0.57)	1.50 (0.58)	1.28 (0.46)
	Values, n (%)	4 (100)	3 (75)	3 (75)	3 (50)	1 (33)	2 (50)	13 (72)
**COVID-19**								
	Values, mean (SD)	1.5 (0.53)	1.25 (0.50)	1.50 (0.58)	1.5 (0.55)	1.00 (0.00)	1.25 (0.50)	1.44 (0.51)
	Values, n (%)	2 (50)	3 (75)	2 (50)	3 (50)	3 (100)	3 (75)	10 (56)
**Drone competence**								
	Values, mean (SD)	1.75 (0.50)	1.75 (0.50)	2.00 (0.00)	2.00 (0.00)	2.00 (0.00)	1.75 (0.50)	1.89 (0.32)
	Values, n (%)	1 (25)	1 (25)	0 (0)	0 (0)	0 (0)	1 (25)	2 (11)
**Medication app competence**								
	Values, mean (SD)	2.00 (0.00)	2.00 (0.00)	2.00 (0.00)	1.5 (0.55)	1.67 (0.57)	1.5 (0.58)	1.83 (0.38)
	Values, n (%)	0 (0)	0 (0)	0 (0)	3 (50)	1 (33)	2 (50)	3 (17)

^a^n has been adjusted by weighting.

### Quantitative Results

#### Bivariate Correlations

Bivariate correlations showed that the more usable (SUS) the technology was, the more the participants were willing to use it (TUI; *r*=0.833). Moreover, the more they found the technology useful (TUI), the more the technology was rated as usable (*r*=0.711; SUS and *r*=0.487; TUI) and the more participants would use the pharmacy drone app (TUI; *r*=0.754). In addition, the intention to use (TUI) the app was positively correlated with curiosity (TUI; *r*=0.550). The more skeptical (TUI) the participants were, the more participants rated the app as unusable (*r*=−0.542; SUS and *r*=−0.479; TUI) and would not be willing to use it (*r*=−0.446; TUI).

Furthermore, the intention to use (TUI) the technology was positively correlated with the perceived task competence (*r*=0.784; first iteration loop) and the need satisfaction of participant’s competence (*r*=0.788; third iteration loop). The more participants felt competent in handling the app in the spheres of task (*r*=0.829; ie, engaging with the app; first iteration loop) and interface (*r*=0.929; ie, interacting with the app; second iteration loop), the higher was perceived usability (SUS). However, the more participants felt competent in task (*r*=0.675) and behavior (*r*=0.875), the more they felt autonomous (ie, the app provided options and participants did not feel under pressure from the app). The more curious the participants were, the more they felt satisfied in psychological needs of competence, autonomy, and relatedness (*r*=0.744). However, the absence of need satisfaction does not equate with the presence of need frustration [17,35]. The results revealed that the more participants rated the app as usable, the lesser they felt autonomy (*r*=−0.792; SUS and *r*=−0.768; TUI) and competence (*r*=−0.751; TUI) frustration. The more they believed the technology would be useful for them, the lesser they felt autonomy frustration (*r*=−0.827). Overall frustration shows a negative correlation with usability (*r*=−0.822; SUS and *r*=−0.799; TUI), usefulness (*r*=−0.923; TUI), and intention to use (*r*=−0.730; TUI). Moreover, the results indicated that autonomy frustration is a relevant marker for overall need frustration (*r*=0.933).

Furthermore, correlations showed that the more time participants needed to solve the task within the app, the less usable the app was rated (*r*=−0.534; SUS), the lesser they were willing to use the app (*r*=−0.429; TUI), the lesser they felt competent (*r*=−0.805), the more skepticism they had (*r*=0.504), and the older the participants were (*r*=0.681). The older the participants were, the more skeptical they were (*r*=.525) and the lesser they believed that the technology was accessible (*r*=−0.510). Female participants showed more technology anxiety (*r*=−0.505), had more interest (*r*=0.497), and felt more related by using the app (*r*=−0.800). The results of 2-tailed *t* tests confirmed these differences between female and male participants (anxiety: t_19_=0.003; interest: t_23=_0.012; relatedness satisfaction: *t*_6_=0.004).

#### TUI Assessment

To test whether anxiety, curiosity, interest, skepticism, usefulness, usability, and accessibility contributed to higher intention to use, we regressed participant’s ratings of these variables on their intention to use and controlled for age, gender, and duration using the app. Results showed that TUI factors such as usefulness, skepticism, and curiosity explain most of the variance in intention to use the pharmacy drone app (*F*_3,17_=21.12; *P*<.001; *R*^2^=0.788; adjusted *R*^2^=0.751; Table 5). Usefulness (β=.499; *P*=.001) and curiosity (β=.376; *P*=.008) were significantly and positively associated with intention to use, whereas skepticism (β=−.397; *P*=.004) was significantly and negatively related with intention to use.

**Table 5:** Hierarchical regression analysis predicting intention to use the technology per the Technology Usage Inventory factors.

Model and predictor		Unstandardized coefficients, B (SE)	Standardized coefficients			*R* ^2^		*R*^2^ change		*F* test (*df*)		*P* value
			β	*P* value								
**1**					0.582		0.560		26.48 (1,19)		<.001	
	Usefulness	14.73 (2.86)	.763	<.001								
**2**					0.676		0.640		18.81 (2,18)		<.001	
	Usefulness	13.03 (2.69)	.675	<.001								
	Skepticism	−9.82 (4.29)	−.319	.03								
**3**					0.788		0.751		21.12 (3,17)		<.001	
	Usefulness	9.638 (2.51)	.499	.001								
	Skepticism	−12.20 (3.66)	−.397	.004								
	Curiosity	6.36 (2.12)	.376	.008								

Descriptively, anxiety, curiosity, interest, usefulness, and accessibility did not vary much between the core group and ad hoc groups, as shown in Table 6. Ad hoc groups appeared to be slightly more skeptical about the pharmacy drone app compared to the core group. This could potentially be attributed to age differences, as those in the ad hoc groups were older than those in the core group, and skepticism was positively correlated with age (Multimedia Appendix 2). Although usability remained at slightly below average in the ad hoc groups, it increased from average to slightly above average in the core group from the first to third iteration. Moreover, the core group showed higher ratings on the intention to use the pharmacy drone app than the ad hoc groups.

**Table 6:** Mean values of the Technology Usage Inventory stanine of the ad hoc and core groups per iteration loop.

		Ad hoc group 1	Ad hoc group 2	Ad hoc group 3	Core group 1	Core group 2	Core group 3
**Psychological factors, mean (SD)^a^**							
	Anxiety	4.75 (1.89)	4.67 (3.05)	5.25 (1.50)	3.50 (1.22)	—^b^	3.00 (1.16)
	Curiosity	7.00 (1.41)	7.67 (0.58)	5.25 (2.36)	7.33 (1.03)	—	7.00 (0.00)
	Interest	5.50 (1.91)	5.25 (1.89)	7.25 (1.50)	6.00 (1.41)	5.67 (1.53)	6.00 (0.82)
	Skepticism	3.75 (1.89)	2.75 (0.96)	3.50 (1.00)	2.83 (0.98)	1.67 (0.58)	2.00 (0.82)
**Technology-specific factors, mean (SD)^a^**							
	Usefulness	8.00 (1.41)	8.75 (0.50)	8.00 (2.00)	8.67 (0.82)	9.00 (0.00)	8.75 (0.50)
	Usability	4.50 (1.91)	4.25 (1.50)	4.50 (0.58)	5.50 (1.05)	6.67 (0.58)	7.00 (0.82)
	Accessibility	7.75 (1.26)	4.50 (1.00)	6.25 (1.26)	7.33 (1.03)	8.67 (0.58)	7.25 (0.96)
Intention to use, mean (SD)^a^		6.75 (1.89)	8.50 (0.58)	5.75 (0.96)	8.50 (0.55)	8.00 (1.00)	8.75 (0.50)

^a^Stanine: 1 to 2, strongly below average; 3 to 4, slightly below average; 5, average; 6 to 7, slightly above average; and 8 to 9, strongly above average [33].

^b^Missing data.

The results of *t* tests showed a higher rating on usability for core group 3 compared with core group 1 (*t*_8_=−2.68; *P*=.03). Moreover, independent samples *t* tests showed a higher anxiety (t_19_=2.88; *P*=.01) as well as a higher skepticism toward the technology (t_23_=2.17; *P*=.04) within the ad hoc group compared with the core group over all iteration loops. Moreover, the core group rated the app more usable than the ad hoc group (t_23_=−3.33; *P*=.003) over all iteration loops. Although descriptive data showed a higher rating on intention to use the pharmacy drone app within the core group (mean 90.36, SD 11.01) than the ad hoc groups (mean 76.00, SD 23.74) over all iteration loops, 2-tailed *t* tests became not significant (*t*_3_=−1.91; *P*=.07).

#### SUS Score

Descriptively, perceived usability (SUS score) decreased between ad hoc group 1 (rated as marginal) and ad hoc group 3 (rated as poor). Within the core group, the usability increased from core group 1 (rated as good) to core group 3 (rated as excellent). However, within the second iteration loop, both groups (ad hoc and core groups) rated the app more usable than during the first and third iteration loops. Moreover, the core group showed higher SUS scores than the ad hoc groups (Table 7). Results of the *t* tests indicated a significant group difference in SUS score between ad hoc group 2 and ad hoc group 3 (*t*_6_=3.35; *P*=.01). The results indicated lower SUS scores of ad hoc group 3 (rated as poor) than ad hoc group 2 (rated as excellent). Furthermore, independent samples *t* tests showed a significant group difference in the SUS score between the ad hoc and core groups over all iteration loops, with higher scores for the core group than the ad hoc groups (t_23_=−2.87; *P*=.004).

**Table 7:** System Usability Score (SUS) scores (0 to 34: very poor; 35 to 49: poor; 50 to 67: marginal; 68 to 79: good; 80 to 89: excellent; and 90 to 100: outstanding [32]) in the ad hoc and core groups per iteration loop.

Group	SUS scores, mean (SD)
Ad hoc group 1	54.38 (23.22)
Ad hoc group 2	80.00 (14.43)
Ad hoc group 3	49.38 (11.25)
Core group 1	75.42 (16.84)
Core group 2	88.33 (10.10)
Core group 3	84.38 (5.54)
Ad hoc group 1 + core group 1	67.00 (21.34)
Ad hoc group 2 + core group 2	83.57 (11.57)
Ad hoc group 1 + core group 3	66.88 (20.43)
All groups	71.60 (19.75)

#### Overall Regressions

When regressing all factors of all usability and psychological needs, the results showed that the SUS usability score as well as the TUI factors such as curiosity and interest explained most of the variance on intention to use the pharmacy drone app (*F*_3,16_=40.27; *P*<.001; *R*^2^=.883; adjusted *R*^2^=.861; Table 8). Usability (β=.845; *P*<.001), curiosity (β=.232; *P*=.02), and interest (β=.195; *P*=.04) were significantly and positively associated with intention to use.

**Table 8:** Hierarchical regression analysis predicting intention to use the technology per the Technology Usage Inventory, System Usability Scale (SUS), Technology-based Experience of Need Satisfaction Task, and Basic Psychological Need Satisfaction and Frustration Scale factors.

Model and predictor		Unstandardized coefficients, B (SE)	Standardized coefficients			*R* ^*2*^		*R*^*2*^ change		*F* test (*df*)		*P* value
			β	*P* value								
**1**					0.789		0.777		67.16 (1,18)		<.001	
	SUS usability	0.999 (0.122)	.888	<.001								
**2**					0.848		0.830		47.31 (2,17)		<.001	
	SUS usability	0.890 (0.115)	.791	<.001								
	Curiosity	4.45 (1.73)	.262	.02								
**3**					0.883		0.861		40.27 (3,16)		<.001	
	SUS usability	0.951 (0.107)	.845	<.001								
	Curiosity	3.94 (1.58)	.232	.02								
	Interest	2.88 (1.31)	.195	.04								

### Qualitative Results

#### First Iteration

**Figure 4:**
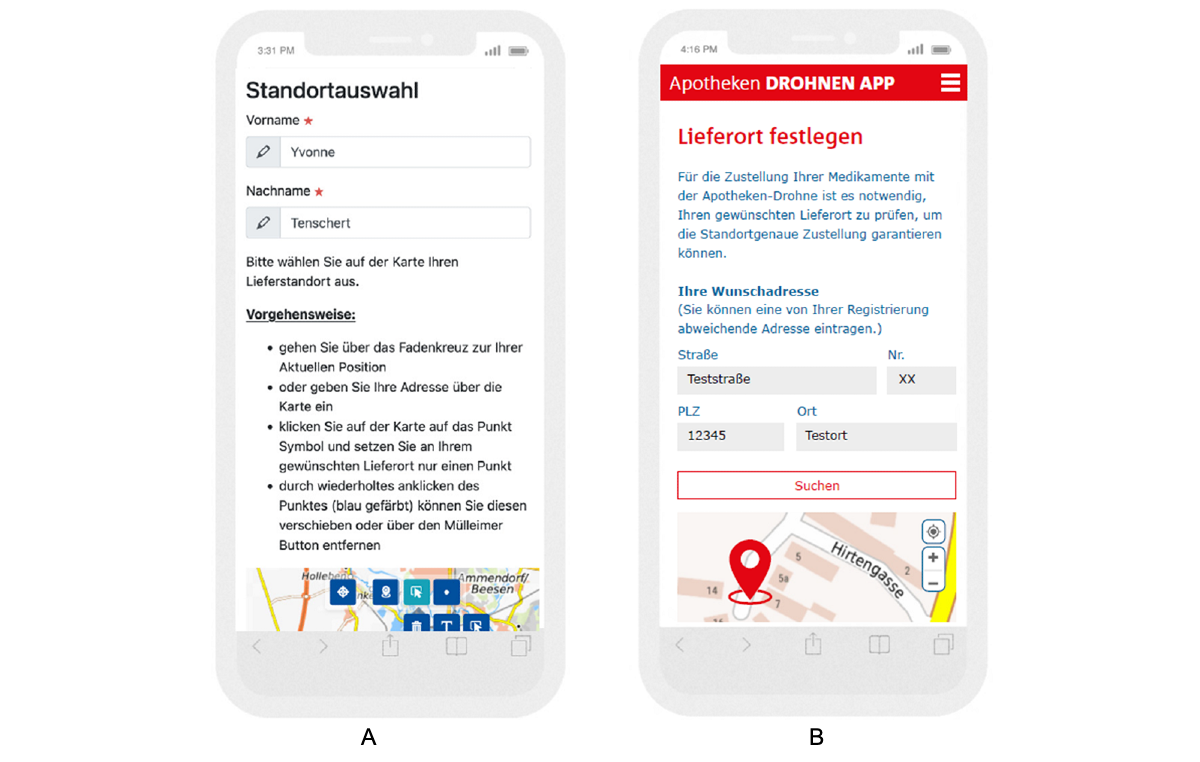
User-centered app design (A) before and (B) after first iteration: delivery location.

**Figure 5:**
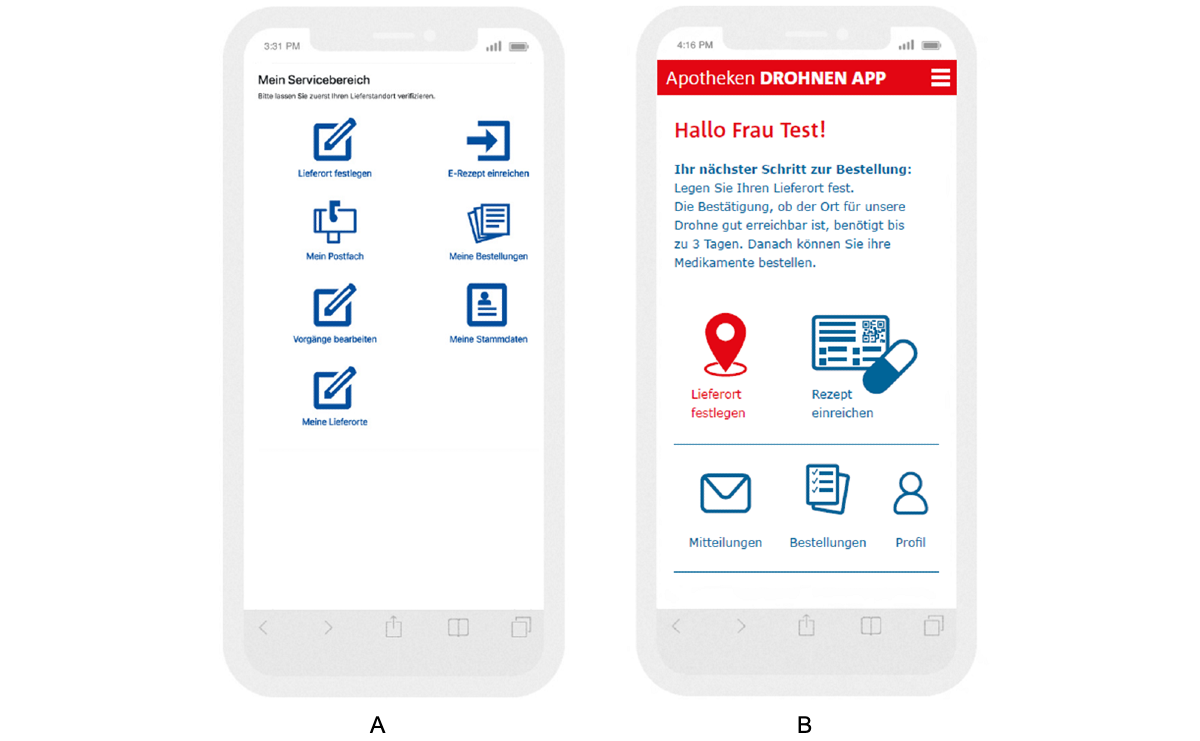
User-centered app design (A) before and (B) after first iteration: symbols.

After the first iteration, the app received a new design according to user’s feedback. Important points after the first iteration were providing more guidance through the app with information about next steps and reasons for doing these steps (eg, the importance of setting the delivery location, clear information about how to choose the delivery location, and more precise symbols; Figures 4 and 5); adaption of conceptualizations (eg, “location” [German: *Standort*] to “delivery point” [German: *Lieferort*]; Figure 4); automatizations (eg, transferring address data automatically to the map); minimizations (eg, reducing symbols within the map and information within each step); communication options (eg, integrating a field to formulate a message to the pharmacist); control features (eg, an order summary); and autonomy options (eg, to upload >1 prescription if necessary). However, participants missed a visualization of password requirements and a preview function of the uploaded recipe. Furthermore, users would rather preview individual pages per click than perform 1-page scrolling. They would also desire information about payment options within the app as well as details about medication availability. This necessitates integration of the interface with the pharmacy’s merchandise management system, which requires additional technical and regulatory administration. A short solution for that was to integrate a comment field at the step of ordering the medication to describe further medication wishes as well as to ask questions to the pharmacist. Moreover, participants emphasized, at this point, the importance of shipment tracking, as Fink et al [27] described in their study. However, the most difficult step participants reported was setting of the delivery location. This was also shown in the amount of support needed while using the app (Multimedia Appendix 3). Although experimenters were instructed to not help participants, at some points the help was necessary so that the participants could finish the task.

#### Second Iteration

After the second iteration, participants in both the core group and the ad hoc group reported that registration was simplified, indicating that it was easy for them to register. Moreover, participants reported that the summary of order was clear, the texts were more comprehensible (core group), and the participants liked the option to upload >1 prescription (ad hoc group). However, participants missed a preview function of the uploaded prescription as a picture and the selection of push messages. Moreover, they suggested highlighting the icon, which is the next step to be clicked on (eg, after setting the delivery location, the icon for submitting the prescription should be highlighted), and highlighting the inbox when a new message has arrived. Important points after the second iteration were still reducing symbols within the map (eg, symbols were reduced to a minimum, and the text describing the symbols was shortened because the participants did not read the instructions above the card); more guidance through the app with information about next steps; and more transparency about how a response will be received from pharmacists (eg, integration of information about how the contact will take place, via mail or telephone), indications on password requirements (such as length, upper and lower case, and special characters), and information about shipment tracking. An important safety-relevant point was that flight slots were not up to date according to the original time. Similar to the first iteration loop, participants reported setting the delivery location as the most difficult step. They would like to have more guidance for the subsequent steps after determination of the delivery location.

#### Third Iteration

The core group reported that the app was more intuitive and had improved compared with the first iteration loop. They mentioned that the automated fill-in of address data in the map as well as the identification that the prescription was successfully uploaded was useful.

Important points after the third iteration were more guidance through a processing status within the app; adaption of conceptualization (eg, “mailbox” [German: *Postfach*] to “messages” [German: *Nachrichten*]); visualization of password requirements (eg, upper and lower case and special characters); automatization features (eg, automatic suggestions such as city when entering the postal code); differentiation (eg, distinction between the delivery and billing address); control (eg, adjustment of the amount of information within the app to control how much information the user wants, which might be configured via profile); push messages instead of emails because participants did not read the texts despite shortening them; a preview function of the uploaded prescription; and design features (eg, it was not clear that scrolling was necessary, thus more guidance is useful through individual pages [click to continue]). They also desired the inbox to be highlighted when new messages had arrived.

Although the app was adapted according to the participant’s feedback, the ad hoc group reported that the step of setting the delivery location remained too complex for them. They mentioned that this step was too bulky, time consuming, and not intuitive despite adjustments such as the reduction of map symbols, providing the most important information, shortening the text, and inserting address data automatically in the map. Participants of the ad hoc group felt lost and helpless during this step. However, during discussions after thinking aloud, participants suggested that the setting of delivery comes from the operator and thus must not be made by users. They only wanted confirmation of the delivery point to ensure its correctness. Excluding this step might decrease the likelihood of user errors.

However, across all iterations, criteria emerged that were repeatedly the focus of the participants’ attention: automatization (ie, easy and fast use for avoiding redundancies), minimization (ie, as little information as possible and as much as necessary), differentiation (ie, clear distinctions), control (ie, options to choose), guiding (ie, concise and understandable instructions supporting guidance through the app), conceptualization (ie, easy and precise language), barrier-free design (ie, uniformity between different steps and intuitive visualizations), and transparency (about disclosures to be made or obtained information).

**Figure 6:**
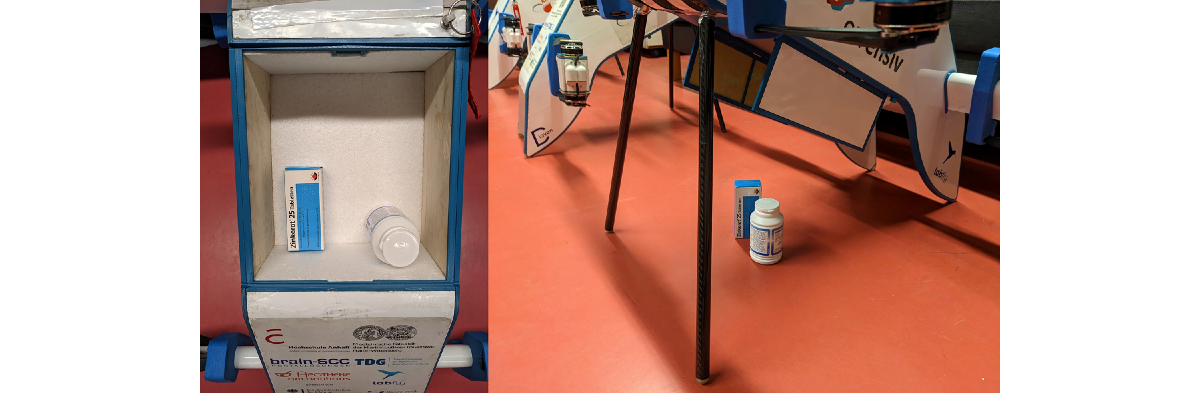
Drone medication ejection.

Although we could not test the handover scenarios, we have made modifications to the drone. Previous results of focus group testing within the ADApp project showed concerns about injuries caused by the drone [27]. Thus, the drone has now been given a flap underneath so that the medications can be dropped by ejection, using a parachute, or using a winch and a landing of the drone can be prevented (Figure 6). Further testing is planned to test different handover scenarios with participants to adapt the handover according to their needs.

## Discussion

### Factors of User Acceptance

This study aimed at investigating factors that are associated with the user acceptance of a drone-based medication delivery by using a mixed methods design to be able to derive practice-relevant orientation points for participatory technology development (for apps and drones).

First, an important point is duration handling with the app. Older participants needed more time to solve the tasks within the app. Furthermore, the longer the process took, the more the usability, intention to use the app, and feelings of competence decreased, while skepticism increased. Therefore, the duration of interaction with a technology appears to be a crucial factor for user acceptance.

Second, psychological factors such as skepticism and curiosity as well as technology-specific factors such as usefulness and usability are related with participants’ intention to use the technology for a drone-based medication delivery. Regression analyses within the TUI factors revealed usefulness, curiosity, and skepticism as significant predictors for intention to use the technology, wherein usefulness explained the highest variance (49.9%), which is consistent with the findings of Güsken et al [47]. This implies a particular relevance of factor usefulness for the development of technologies in health care, especially in drone-based medication delivery. With the help of the TUI questionnaire, we can conclude that curiosity and skepticism affect user acceptance. The more the users were curious about the pharmacy drone app and the less skeptical they were, the more the users were willing to use and interact with the technology. This is consistent with the findings of Eißfeld et al [6] who found that a positive attitude toward drones and a general technical interest are related to improved information about it.

Third, basic human needs according to Ryan and Deci [16,17] and Deci and Ryan [18]also play an important role. Results of this study showed that the more participants perceived competence in handling the app, the more they are willing to use the technology and the more they rated the app as usable. This implies that, although competence satisfaction in all iteration loops was related with usability and the intention to use, autonomy and relatedness were not related. Nevertheless, results showed that the more participants felt competent, the more they felt autonomous. Moreover, the basic psychological needs (competence, autonomy, and relatedness) were positively correlated with curiosity. In addition, the lesser participants felt frustrated on psychological needs, the higher they rated the usability, usefulness, and intention to use. Thus, following the Basic Psychological Need Theory [17], the more the interaction with the system satisfies basic psychological needs, the more the users will engage with a technology. Following the METUX model, an increase in autonomy increases engagement and an increase in competence increases motivation of using the app, which is in accordance with the results of this study [15]. Interestingly, while competence and autonomy appear to be significant factors in explaining differences in intention to use and usability, relatedness does not play a role, despite focus group discussions emphasizing the importance of communication and consultation features within such technology [27]. One reason for this result might be the nature of the study task. Although participants used the app in a simulated context, communication aspects did not play a role in this developing step of technology. Therefore, supply studies in real-life contexts are necessary to test the impact of psychological and technological factors on real-life complex problems, which cannot be fully investigated in a simulated context such as in this study, where, for example, relatedness might hold greater significance in real-life scenarios than in simulations, as increased relatedness could potentially enhance overall well-being [15].

Fourth, overall regression analyses showed that usability, curiosity, and interest explain most of the variance on intention to use the pharmacy drone app, wherein usability showed the strongest effect (84.5%). This means, when adding all factors in one model, usability becomes the strongest predictor for intention to use the pharmacy drone app. Similar to other studies, this study found evidence for the importance of usability in using a technology [1,5]. This means that better usability of a technology leads to higher acceptance [12,33]. However, this study used a very small sample group, and the statistical results should be considered carefully. In conclusion, studies with higher sample sizes are necessary.

Taken together, the intention to use a drone-based medication delivery system is a comprehensive construct that is based on a large number of underlying, explanatory factors. This study showed that usability, curiosity, and interest had a considerable impact on intention to use, wherein usefulness, skepticism, and competence also played an important role. The failure to actively involve users in technology development can thus result in insufficient addressing of profession- and person-specific needs, thus resulting in a lack of intention to use the technology. For successful technology development, it is therefore crucial to develop an understanding of the necessary characteristics of health care technologies and to identify the determinants that ensure a high level of acceptance for improving the current supply situation [47].

In accordance with the previous scoping review [7], user feedback was collected iteratively and focused on user experience. The TA method [38] used in this process provided valuable insights that were taken into account when developing the app. In this way, important changes to the app were successfully implemented by user request such as a reduced design and automatic fill-in aids. It was found that communication with the dispatcher and shipment tracking are very important to users, which is consistent with the assumptions from the scoping review [7], and this also led to further adjustments. The changes made could be verified in the further iterations; for example, it turned out that the revision for the definition of the delivery location was not helpful: the process was adjusted based on participant feedback with more information, but this step remained too complex. In the third iteration, it became clear that the required texts were not being read at this point, leading to the ultimate decision to omit this step altogether as participants indicated that they only wanted to confirm the delivery location.

### Differences in User Acceptance

A second purpose of this study was to assess group differences between a core group and ad hoc groups in user acceptance.

The results of this study indicated the importance of an ad hoc group in an iterative, cocreative process. Although within the core group, intention to use (TUI) was similar over all iteration loops (strongly above average), within the ad hoc group, intention to use varied from slightly above average to strongly above average to average. Although within the core group, the usability of the app slightly increased from “average resp. good” to “slightly above average resp. excellent,” the usability within the ad hoc group decreased from marginal to poor (SUS) and remained slightly above average (TUI). Results of *t* tests of both questionnaires showed a significant group difference between the ad hoc and core groups, with higher ratings in perceived usability for the core group. This suggests that repeated measurements induce a shift in the interpretation of test scores, potentially biasing the measurement of change [26]. Thus, this study shows that repeated measures with the same sample group might change their attitude, expectations, and behavior in dealing with the technology, which changed their ratings on usability. The core group then tended to evaluate the app better than the ad hoc group because they were not unfamiliar with dealing with the app. Thus, for naive users, the app is just not intuitive and easy enough to use. An additional explanation for this result is that the ad hoc groups were more anxious and skeptical than the core group, wherein a higher skepticism was found to be related to lower ratings on usability [47]. However, within the usability score (SUS), data showed an increase from the first to second iteration and a decrease from the second to third iteration in both groups, wherein the ad hoc groups rated the pharmacy drone app as less usable compared with the core group. One reason for the decrease from the second to third iteration could be the more complex setting during the third iteration: the participants had to run through the entire process from registration, setting the delivery location, and ordering the medication to receiving the medication. Meanwhile, the core group showed a learning effect and maybe thus rated the pharmacy drone process to be more usable from the first iteration to second iteration, and the ad hoc groups could stumble because of the complexity.

### Limitations

This study shows for the first time the importance of ad hoc groups as a control group while developing and evaluating a technology in a user-centered design. When interpreting the results of this study, several methodological limitations must be considered. In terms of age and gender distribution, the sample can be classified as unrepresentative owing to the small number of participants. This is particularly evident in the statistical evaluation. Nevertheless, there is a basic tendency toward a clear effect, which is evident despite the small number of samples. The participants had a basic interest in new topics and in the topic itself. Although the risk of “positive selection” cannot be completely ruled out, it is not seen because the topic of drone-assisted medication delivery was largely unknown. Thus, the perspectives of participants who consistently reject technical systems in the context of care and delivery were as poorly represented as those who chose not to participate in the surveys for other reasons. Reasons for this could include a general dismissive attitude toward additional effort owing to time resources, heavy workloads, or other thematic priorities.

However, it can be concluded that the results obtained to assess the acceptance of the drone app for utility purposes have revealed important insights regarding technical development and its practical use. In this context, the findings exhibit similarities to surveys conducted for other target groups in health care.

### Conclusions

The study highlights the significance of understanding the essential attributes of health care technologies and the factors that lead to their acceptance in improving the current supply situation. It offers valuable insights for practitioners to develop participatory technologies and recommends ad hoc groups as a complementary approach to control the process of a user-centered design. However, larger samples and real-world contexts are required to confirm these findings.

## Appendix

Multimedia Appendix 1 Examples of criteria.

Multimedia Appendix 2 Bivariate correlations between System Usability Scale (SUS), Technology Usage Inventory (TUI), Technology-based Experience of Need Satisfaction (TENS) Task, TENS Interface, and Basic Psychological Need Satisfaction and Frustration Scale (BPNSFS).

Multimedia Appendix 3 Support required per task.

## Abbreviations

ADApp: Apotheken-Drohnen-App
BPNSFS: Basic Psychological Need Satisfaction and Frustration Scale
METUX: Motivation, Engagement, Thriving in User Experience
SUS: System Usability Scale
TAM: technology acceptance model
TA: think-aloud
TENS: Technology-based Experience of Need Satisfaction
TUI: Technology Usage Inventory

## References

[1] Hiebert B, Nouvet E, Jeyabalan V, Donelle L (2020). The application of drones in healthcare and health-related services in North America: a scoping review. Drones.

[2] Saponi M, Borboni A, Adamini R, Faglia R, Amici C (2022). Embedded payload solutions in UAVs for medium and small package delivery. Machines.

[3] Sabino H, Almeida RV, Moraes LB, Silva WP, Guerra R, Malcher C, Passos D, Passos FG (2022). A systematic literature review on the main factors for public acceptance of drones. Technol Soc.

[4] Rave A, Fontaine P, Kuhn H Drone network design for emergency resupply of pharmacies and ambulances. https://papers.ssrn.com/sol3/papers.cfm?abstract_id=4569199.

[5] Rejeb A, Rejeb K, Simske S, Treiblmaier H (2021). Humanitarian drones: a review and research agenda. Internet Things.

[6] Eißfeldt H, Vogelpohl V, Stolz M, Papenfuß A, Biella M, Belz J, Kügler D (2020). The acceptance of civil drones in Germany. CEAS Aeronaut J.

[7] Stephan F, Reinsperger N, Grünthal M, Paulicke D, Jahn P (2022). Human drone interaction in delivery of medical supplies: a scoping review of experimental studies. PLoS One.

[8] Sham R, Siau CS, Tan S, Kiu DC, Sabhi H, Thew HZ, Selvachandran G, Quek SG, Ahmad N, Ramli MH (2022). Drone usage for medicine and vaccine delivery during the COVID-19 pandemic: attitude of health care workers in rural medical centres. Drones.

[9] Milchrahm E (2002). Entwicklung eines Modells zur Akzeptanzproblematik von Informationstechnologie. Proceedings of the Information und Mobilität, Optimierung und Vermeidung von Mobilität durch Information.

[10] Krey M, Seiler R (2019). Usage and acceptance of drone technology in healthcare: exploring patients and physicians perspective. Proceedings of the 52nd Hawaii International Conference on System Sciences.

[11] Simon B (2001). E-Learning an Hochschulen. Gestaltungsräume und Erfolgsfaktoren von Wissensmedien.

[12] Davis FD (1989). Perceived usefulness, perceived ease of use, and user acceptance of information technology. MIS Q.

[13] Venkatesh V, Bala H (2008). Technology Acceptance Model 3 and a research agenda on interventions. Decis Sci.

[14] Venkatesh V, Davis FD (2000). A theoretical extension of the technology acceptance model: four longitudinal field studies. Manag Sci.

[15] Peters D, Calvo RA, Ryan RM (2018). Designing for motivation, engagement and wellbeing in digital experience. Front Psychol.

[16] Ryan RM, Deci EL (2000). Intrinsic and extrinsic motivations: classic definitions and new directions. Contemp Educ Psychol.

[17] Ryan RM, Deci EL (2017). Self-Determination Theory: Basic Psychological Needs in Motivation, Development, and Wellness.

[18] Deci EL, Ryan RM (1985). Intrinsic Motivation and Self-Determination in Human Behavior.

[19] Jockisch M, Bandow G, Holzmüller H (2010). Das ist gar kein Modell!. Das Technologieakzeptanzmodell.

[20] Farao J, Malila B, Conrad N, Mutsvangwa T, Rangaka MX, Douglas TS (2020). A user-centred design framework for mHealth. PLoS One.

[21] Risling TL, Risling DE (2020). Advancing nursing participation in user-centred design. J Res Nurs.

[22] Schnall R, Rojas M, Bakken S, Brown W, Carballo-Dieguez A, Carry M, Gelaude D, Mosley JP, Travers J (2016). A user-centered model for designing consumer mobile health (mHealth) applications (apps). J Biomed Inform.

[23] Nilsen W, Kumar S, Shar A, Varoquiers C, Wiley T, Riley WT, Pavel M, Atienza AA (2012). Advancing the science of mHealth. J Health Commun.

[24] McCurdie T, Taneva S, Casselman M, Yeung M, McDaniel C, Ho W, Cafazzo J (2012). mHealth consumer apps: the case for user-centered design. Biomed Instrum Technol.

[25] Altman M, Huang TT, Breland JY (2018). Design thinking in health care. Prev Chronic Dis.

[26] Oort FJ, Visser MR, Sprangers MA (2009). Formal definitions of measurement bias and explanation bias clarify measurement and conceptual perspectives on response shift. J Clin Epidemiol.

[27] Fink F, Paulicke D, Grünthal M, Jahn P (2023). "Of course, drones delivering urgent medicines are necessary. But I would not use them until…" Insights from a qualitative study on users' needs and requirements regarding the use of medical drones. PLoS One.

[28] Brooke J (1996). SUS: a 'quick and dirty' usability scale. Usability Evaluation In Industry.

[29] Brooke J (2013). SUS: a retrospective. J Usab Stud.

[30] Hyzy M, Bond R, Mulvenna M, Bai L, Dix A, Leigh S, Hunt S (2022). System usability scale benchmarking for digital health apps: meta-analysis. JMIR Mhealth Uhealth.

[31] Sauro J (2011). A Practical Guide to the System Usability Scale: Background, Benchmarks & Best Practices.

[32] Bangor A, Kortum P, Miller J (2009). Determining what individual SUS scores mean: adding an adjective rating scale. J Usab Stud.

[33] Kothgassner OD, Felnhofer A, Hauk N, Kastenhofer E, Gomm J, Ryspin-Exner I (2012). TUI: Technology Usage Inventory.

[34] Febretti A, Garzotto F (2009). Usability, playability, and long-term engagement in computer games. Proceedings of the CHI '09 Extended Abstracts on Human Factors in Computing Systems.

[35] Chen B, Vansteenkiste M, Beyers W, Boone L, Deci EL, Van der Kaap-Deeder J, Duriez B, Lens W, Matos L, Mouratidis A, Ryan RM, Sheldon KM, Soenens B, Van Petegem S, Verstuyf J (2015). Basic psychological need satisfaction, need frustration, and need strength across four cultures. Motiv Emot.

[36] Heissel A, Pietrek A, Flunger B, Fydrich T, Rapp MA, Heinzel S, Vansteenkiste M (2018). The validation of the German basic psychological need satisfaction and frustration scale in the context of mental health. Eur J Health Psychol.

[37] Gunzenhauser C, Karbach J, Saalbach H (2019). Function of verbal strategies in monolingual vs. bilingual students’ planning performance: an experimental approach. Cognit Dev.

[38] Jaspers MW, Steen T, van den Bos C, Geenen M (2004). The think aloud method: a guide to user interface design. Int J Med Inform.

[39] Fonteyn ME, Kuipers B, Grobe SJ (2016). A description of think aloud method and protocol analysis. Qual Health Res.

[40] Johnson WR, Artino AR Jr, Durning SJ (2022). Using the think aloud protocol in health professions education: an interview method for exploring thought processes: AMEE Guide No. 151. Med Teach.

[41] Roberts JP, Fisher TR, Trowbridge MJ, Bent C (2016). A design thinking framework for healthcare management and innovation. Healthc (Amst).

[42] Ericsson KA, Ericsson KA, Charness N, Feltovich PJ, Hoffman RR (2006). Protocol analysis and expert thought: concurrent verbalizations of thinking during experts’ performance on representative tasks. The Cambridge Handbook of Expertise and Expert Performance.

[43] Berelson B (1952). Content Analysis in Communication Research.

[44] Winsler A, Fernyhough C, McClaren EM, Way E (2005). Private speech coding manual. Unpublished manuscript. George Mason University.

[45] Cohen J (1960). A coefficient of agreement for nominal scales. Educ Psychol Measure.

[46] Landis JR, Koch GG (1977). The measurement of observer agreement for categorical data. Biometrics.

[47] Güsken SR, Frings K, Zafar F, Saltan T, Fuchs-Frohnhofen P, Bitter-Krahe J (2021). Einflussfaktoren auf die Nutzungsintention von Pflegekräften zur Verwendung digitaler Technologien in der ambulanten Pflege. Fallstudie zur Einführung eines Sensortexils. Z Arbeitswiss.

