# Supplementary material for: Identifying Factors of User Acceptance of a Drone-Based Medication Delivery: User-Centered Design Approach
Source: JMIR Hum Factors. 2024 Apr 30;11:e51587. doi: 10.2196/51587 (PMC11094598; doi:10.2196/51587)
Supplement: Multimedia Appendix 1 [file humanfactors_v11i1e51587_app1.docx]

**Multimedia Appendix 1.** Examples of criteria.

| **criteria** | **concepts** | **example** |
| --- | --- | --- |
|  |  |  |
| safety | uncertainty | uncertainty what happens after specify delivery |
| risk in the delivery | proposal; uncertainty | necessity of shipment tracking; flight slots not up to date |
| optimization potential | value | pop-up with „welcome“ prevents scrolling |
| outside the capabilities | value | lack of authorization to access cell phone camera |
