# Supplementary material for: Identifying Factors of User Acceptance of a Drone-Based Medication Delivery: User-Centered Design Approach
Source: JMIR Hum Factors. 2024 Apr 30;11:e51587. doi: 10.2196/51587 (PMC11094598; doi:10.2196/51587)
Supplement: Multimedia Appendix 2 [file humanfactors_v11i1e51587_app2.docx]

**Multimedia Appendix 2.** Bivariate Correlations between SUS, TUI, TENS Task, TENS Interface, and BPNSFS.

| **Measures** | | 1 | 2 | 3 | 4 | 5 | 6 | 7 | 8 | 9 | 10 | 11 | 12 | 13 | 14 | 15 | 16 | 17 | 18 | 19 | 20 | 21 | 22 | 23 | 24 | 25 | 26 |
| --- | --- | --- | --- | --- | --- | --- | --- | --- | --- | --- | --- | --- | --- | --- | --- | --- | --- | --- | --- | --- | --- | --- | --- | --- | --- | --- | --- |
| **SUS** | | | | | | | | | | | | | | | | | | | | | | | | | | | |
|  | 1. score | - | - | - | - | - | - | - | - | - | - | - | - | - | - | - | - | - | - | - | - | - | - | - | - | - | - |
| **TUI** | | | | | | | | | | | | | | | | | | | | | | | | | | | |
|  | 2. technology anxiety | ns. | - | - | - | - | - | - | - | - | - | - | - | - | - | - | - | - | - | - | - | - | - | - | - | - | - |
|  | 3. curiosity | ns. | ns. | - | - | - | - | - | - | - | - | - | - | - | - | - | - | - | - | - | - |  | - | - | - | - | - |
|  | 4. interest | ns. | -.469* | ns | - | - | - | - | - | - | - | - | - | - | - | - | - | - | - | - | - | - | - | - | - | - | - |
|  | 5. usability | .626*** | ns. | ns. | ns. | - | - | - | - | - | - | - | - | - | - | - | - | - | - | - | - | - | - | - | - | - | - |
|  | 6. usefulness | .711*** | ns. | ns. | ns. | .487* | - | - | - | - | - | - | - | - | - | - | - | - | - | - | - | - | - | - | - | - | - |
|  | 7. skepticism | -.542** | ns. | ns. | ns. | -.479* | ns. | - | - | - | - | - | - | - | - | - | - | - | - | - | - | - | - | - | - | - | - |
|  | 8. accessibility | ns. | ns. | ns. | ns. | ns. | ns. | -.647*** | - | - | - | - | - | - | - | - | - | - | - | - | - | - | - | - | - | - | - |
|  | 9. intention to use | .833*** | ns. | .550** | ns. | ns. | .754*** | -.448* | ns. | - | - | - | - | - | - | - | - | - | - | - | - | - | - | - | - | - | - |
| **TENS Task (first iteration loop)** | | | | | | | | | | | | | | | | | | | | | | | | | | | |
|  | 10. competence | .829** | ns. | ns. | ns. | ns. | ns. | ns. | ns. | .784* | - | - | - | - | - | - | - | - | - | - | - | - | - | - | - | - | - |
|  | 11. autonomy | ns. | ns. | ns. | ns. | ns. | ns. | ns. | ns. | ns. | .675* | - | - | - | - | - | - | - | - | - | - | - | - | - | - | - | - |
|  | 12. relatedness | ns. | ns. | ns. | ns. | ns. | ns. | ns. | ns. | ns. | ns. | ns. | - | - | - | - | - | - | - | - | - | - | - | - | - | - | - |
| **TENS Interface (second iteration loop)** | | | | | | | | | | | | | | | | | | | | | | | | | | | |
|  | 13. competence | .929** | ns. | ns. | ns. | ns. | ns. | ns. | ns. | ns. | ns. | ns. | ns. | - | - | - | - | - | - | - | - | - | - | - | - | - | - |
|  | 14. autonomy | ns. | ns. | ns. | ns. | ns. | ns. | ns. | ns. | ns. | ns. | ns. | ns. | ns. | - | - | - | - | - | - | - | - | - | - | - | - | - |
|  | 15. relatedness | ns. | ns. | ns. | ns. | ns. | ns. | ns. | ns. | ns. | ns. | ns. | ns. | ns. | ns. | - | - | - | - | - | - | - | - | - | - | - | - |
| **BPNSFS satisfaction (third iteration loop)** | | | | | | | | | | | | | | | | | | | | | | | | | | | |
|  | 16. competence | ns. | ns. | ns. | ns. | ns. | ns. | ns. | .795* | .788* | ns. | ns. | ns. | ns. | ns. | ns. | - | - | - | - | - | - | - | - | - | - | - |
|  | 17. autonomy | ns. | ns. | ns. | ns. | ns. | ns. | ns. | ns. | ns. | ns. | ns. | ns. | ns. | ns. | ns. | .875** | - | - | - | - | - | - | - | - | - | - |
|  | 18. relatedness | ns. | .745* | ns. | ns. | ns. | ns. | ns. | ns. | ns. | ns. | ns. | ns. | ns. | ns. | ns. | ns. | ns. | - | - | - | - | - | - | - | - | - |
|  | 19. overall | ns. | ns. | .744* | ns. | ns. | ns. | ns. | ns. | ns. | ns. | ns. | ns. | ns. | ns. | ns. | ns. | ns. | ns | - | - | - | - | - | - | - | - |
| **BPNSFS frustration (third iteration loop)** | | | | | | | | | | | | | | | | | | | | | | | | | | | |
|  | 20. competence | ns. | ns. | ns. | ns. | -.751* | ns. | ns. | ns. | ns. | ns. | ns. | ns. | ns. | ns. | ns. | ns. | ns. | ns. | ns. | - | - | - | - | - | - | - |
|  | 21. autonomy | -.792* | ns. | ns. | ns. | -.768* | -.827* | ns. | ns. | ns. | ns. | ns. | ns. | ns. | ns. | ns. | ns. | ns. | ns. | ns. | ns | - | - | - | - | - | - |
|  | 22. relatedness | ns. | ns. | ns. | ns. | ns. | ns. | ns. | ns. | ns. | ns. | ns. | ns. | ns. | ns. | ns. | ns. | ns. | ns. | ns. | ns. | ns | - | - | - | - | - |
|  | 23. overall | -.822* | ns. | ns. | ns. | -.799* | -.923** | ns. | ns. | -.730* | ns. | ns. | ns. | ns. | ns. | ns. | ns. | ns. | ns. | ns. | ns. | .933*** | ns | - | - | - | - |
| **Demographics** | | | | | | | | | | | | | | | | | | | | | | | | | | | |
|  | 24. age | ns | ns | ns | ns | ns | ns | .525** | -.510** | ns | ns | ns | ns | ns | ns | ns | ns | ns | ns | ns | ns | ns | ns | ns | - | - | - |
|  | 25. gender | ns | -.505* | ns | .497* | ns | ns | ns | ns | ns | ns | ns | ns | ns | ns | ns | ns | ns | -.800* | ns | ns | ns | ns | ns | ns | - | - |
|  | 26. duration | -.534** | ns | ns | ns | ns | ns | .504* | ns | -.429* | ns | ns | ns | ns | ns | ns | -.805* | ns | ns | ns | ns | ns | ns | ns | .681** | ns | - |

Note: *p < .05.; **p < .01.; ***p < .001. ns. = not significant. gender: 1 = female. 2 = male
