# Supplementary material for: Identifying Factors of User Acceptance of a Drone-Based Medication Delivery: User-Centered Design Approach
Source: JMIR Hum Factors. 2024 Apr 30;11:e51587. doi: 10.2196/51587 (PMC11094598; doi:10.2196/51587)
Supplement: Multimedia Appendix 3 [file humanfactors_v11i1e51587_app3.docx]

**Multimedia Appendix 3.** Support required per task.

|  | **ADHOC1** | **CORE1** | **ALL1** |
| --- | --- | --- | --- |
| Task 1: registration | 5 (13.9%) | 5 (20,8%) | 10 (16.7%) |
| Task 2: set delivery location | 18 (50%) | 12 (50%) | 30 (50%) |
| Task 3: submitting prescription | 13 (36.1%) | 7 (29.2%) | 20 (33.3%) |
|  | **ADHOC2** | **CORE2** | **ALL2** |
| Task 1: registration | 4 (23.5%) | 0 | 4 (19.1%) |
| Task 2: set delivery location | 8 (47.1%) | 2 (50%) | 10 (47.6) |
| Task 3: submitting prescription | 5 (29.4%) | 2 (50%) | 7 (33.3%) |
|  | **ADHOC3** | **CORE3** | **ALL3** |
| Task 1: registration | 3 (11.5%) | 2 (33.3%) | 5 (15.6%) |
| Task 2: set delivery location | 14 (57.7%) | 1 (16.7%) | 16 (50%) |
| Task 3: submitting prescription | 8 (30.8%) | 3 (50%) | 11 (34.4%) |
